# Study protocol for “MOVEdiabetes”: a trial to promote physical activity for adults with type 2 diabetes in primary health care in Oman

**DOI:** 10.1186/s12889-016-3990-0

**Published:** 2017-01-06

**Authors:** Thamra S. Alghafri, Saud M. Alharthi, Yahya M. Al-Farsi, Angela M. Craigie, Maureen Mcleod, Annie S. Anderson

**Affiliations:** 1 Directorate General of Health Services, Ministry of Health, PO Box 2723, , Postal Code 112 Muscat, Oman; 2 Department of Family Medicine and Public Health, College of Medicine and Health Sciences, Sultan Qaboos University, Muscat, Oman; 3 Centre for Public Health Nutrition Research, University of Dundee, Ninewells Hospital and Medical School, Dundee, Scotland

**Keywords:** Physical activity, Type 2 diabetes, Primary health care, MOVEdiabetes, Intervention, Oman

## Abstract

**Background:**

Benefits of physical activity in the management of diabetes are well documented. However, evidence on the effectiveness of interventions integrating physical activity in diabetes care is sparse especially in the countries of the Gulf Cooperation Council. The results from this study will increase our understanding of the use of multi-component interventions aimed at increasing physical activity levels in inactive adults with type 2 diabetes in primary health care in Oman.

**Methods/design:**

The study is a one year 1:1 cluster randomized controlled trial of the MOVEdiabetes programme (intervention) versus usual care in eight primary health care centres in Oman. The MOVEdiabetes programme utilizes face to face physical activity consultations promoting 150 min of moderate to vigorous physical activity per week (≥600MET-mins/week), pedometers to self-monitor step counts and monthly telephone WhatsApp messages for follow up support. Inactive adults with type 2 diabetes and no contraindication to physical activity will be recruited over a two months period, and followed up for 12 months. To demonstrate a 50% between group difference in physical activity levels (MET-mins/week) over 12 months, (at a power of 80%, and significance level of 5%), 128 participants would be required to complete the study (64 in each arm). Based on a drop-out rate of 20%, 154 participants would require to be recruited (77 in each arm). Assuming a recruitment rate of 70%, 220 potential eligible participants would need to be approached. The primary outcome is change in levels of physical activity measured by the Global Physical Activity Questionnaire. In addition, accelerometers will be used in a sub group to objectively assess physical activity. Secondary outcomes include changes in metabolic and cardiovascular biomarkers, change in self-reported health, social support, self-efficacy for physical activity, and perceived acceptability of the program. All intervention delivery and support costs will be monitored.

**Discussion:**

This study will contribute to the evidence on the feasibility, cultural acceptability and efficacy of interventional approaches for increasing physical activity in primary care for persons with type 2 diabetes in Oman.

**Trial registration:**

International Standard Randomised Controlled Trials No: ISRCTN14425284. Registered 12 April 2016.

## Background

The International Diabetes Federation (IDF) estimates that 8.3% of people globally have diabetes (382 million), 90% of which is type 2 diabetes (T2D) and the numbers affected are expected to increase by 55% (to 592 million) by 2035 [1]. In countries of the Middle East and North Africa (MENA) region, the impact of diabetes is a major concern, especially in the Arab Gulf countries which have the highest prevalence of diabetes in the world [2].

Oman, similar to the other high income countries of the Gulf Cooperation Council (GCC) [3], has gone through rapid economic development leading to high energy dense diets and sedentary lifestyles [4]. Diabetes prevalence in Oman increased from 8.3% in 1991 to 11.6% in 2000 and 12.3% in 2008 and recent estimates are in the order of 14.2% [1, 5]. Oman and other GCC countries are facing similar challenges related to management of diabetes and the IDF has estimated that the MENA will have a 96% increase in the number of people with diabetes by 2035 [1, 6].

The evidence around the impact of physical activity (PA) on both the prevention and management of T2D is well documented [7, 8]. To reach the clinical benefits of PA, the World Health Organization (WHO) recommends at least 150 min of moderate-intensity PA OR 75 min of vigorous-intensity PA per week, which equates to an equivalent combination of moderate- and vigorous-intensity PA achieving at least 600 MET-min/week. Meeting physical activity recommendations has been shown to increase insulin sensitivity, lower blood sugar levels, reduce body fat and improve physical condition [9].

Based on the best-available data derived from subjective PA measurement tools (e.g. Global Physical Activity Questionnaire (GPAQ), International Physical Activity Questionnaire (IPAQ-Short) and locally developed questionnaires), the population estimate for adults meeting WHO PA recommendations in Arab countries is around 40% for men and 27% for women [10]. A regional study in Sur (north-east coast of Oman), using GPAQ, showed the highest levels of physical inactivity were in leisure time (55%) and the median sitting time was about two hours/day [11].

While in western countries (USA) it has been reported that over 60% of patients with T2D don’t meet the recommended levels of PA [12], consolidated evidence demonstrating PA levels in populations with T2D in Arabic countries is scarce. In Oman, the national health survey 2008 [5] reported that 15% of adults with T2D were physically active based on international recommendations (150 min per week) while in Lebanon only 10% of adults with T2D were reported to reach the recommended levels of PA versus 23% with inadequate levels [13].

Evidence on the best PA interventions for patients with T2D in primary care worldwide is unclear. Interventions differed by settings (primary care vs community), methods (consultations vs exercise sessions), and duration (short-term vs long-term). PA consultations and exercise sessions linked to theories of behaviour change seem to significantly improve activity levels for patients with T2D [14]. Additionally, technology bound interventions and the use of motivational tools, such as pedometers, have been consistently recommended in interventional studies [15, 16]. Walking interventions have also shown significant positive effects in lowering glycated haemoglobin levels (HbA1c) and improved diabetes health outcomes [17]. However, there is still a gap in the evidence on the best methods, intervention components and intervention intensity that would be most effective in increasing long-term PA in the primary care setting for persons with T2D [14, 18].

Almost all interventions have been carried out in non-Arabic speaking countries hence, evidence for culturally sensitive PA interventions is warranted. Based on these gaps in knowledge, this study aims to explore uncertainties about translating existing evidence from western settings to local clinical settings in Oman whilst taking account of cultural boundaries.

## Methods

### Primary objective

The primary objective is to evaluate the impact of a multi-component intervention (MOVEdiabetes) which aims to achieve 150 min of moderate to vigorous physical activity/week (≥600MET-mins/week) in inactive adults with T2D attending primary health care facilities in Oman.

### Secondary objectives

The secondary objectives of the study are:

- Estimate the impact of MOVEdiabetes programme on cardio-metabolic risk factors.
- To evaluate the impact of utilizing a common telephone application, WhatsApp, as an intervention reminder and follow up tool.
- To examine the acceptability of the intervention (content, delivery and aims) to the participants and project officers (health care providers).
- To assess the practical issues (including costs) that could challenge or assist programme delivery and roll out.

## Study design/location/recruitment

### Randomisation

The study is a 1 year 1:1 cluster randomized controlled trial of the MOVEdiabetes intervention versus usual care. A cluster randomization design was used to minimize between group contamination by having the two groups (intervention and comparison) from independent health centres. Group allocation will be generated using a random numbers table generated in SPSS v21 by an independent statistician in Oman Ministry of Health. All health centres (8) will be randomised to deliver either the intervention (*n* = 4) or usual care (*n* = 4). Health centres will be informed of their allocation verbally by the project investigator and will receive an envelope containing invitation letter and project materials.

### Recruitment

To ensure that intervention delivery is institutionalized within routine diabetes care in the selected health centres, three project officers will be recruited at each side (*n* = 24) from existing health care providers (doctors/nurses/dieticians/health educators). Project officers will receive study specific training on the recruitment procedures, screening the participants, recording outcome measurements, and delivering the MOVEdiabetes intervention in intervention health centres (IHC).

All eligible patients attending their routine diabetes clinics in the selected health centers will be informed about the study by the project officers and invited to participate in the study. Interested patients will be screened for physical inactivity using the Scottish Physical Activity Screening Questionnaire (Scot-PASQ) (http://www.paha.org.uk/Resource/scottish-physical-activity-screening-question-scot-pasq﻿).

- **Inclusion criteria**
  - Adults aged 18 to 60 years
  - Diagnosed with type 2 diabetes
  - Attending health centres for at least six months previously for diabetes care
  - Assessed by project officer as having inactive behaviour
  - No contraindication to physical activity
  - Able to speak and read Arabic
  - Willing and able to provide written informed consent to the study
- **Exclusion criteria**
  Patients with:
  - Type 1 diabetes
  - A history of myocardial infarction in the previous 6 months
  - A serum creatinine >140 mmol/L (from previous recorded readings in the electronic health information system)
  - Diabetic foot ulcers or at high risk of ulcer (severe peripheral neuropathy)
  - Repeated hypoglycaemia or severe hypoglycaemia in previous 12 months
  - No internet access for WhatsApp
  - Physical activity > 150 min per week

## Recruitment

Recruitment will take place over a 2 month period (8 weeks). Inactive patients fulfilling the inclusion criteria will be provided with a participant information sheet about the study by the project officers and a subset of patients (40%) showing interest will be offered an accelerometer to be worn for a week prior to their measurement visits to validate the GPAQ results and as a primary measurement tool to evaluate the change in METS-mins/week, sitting time and step counts. Initially all participants (*n* = 220) will be offered accelerometers until the required numbers are reached in all eight health center (*n* = 11). Subsequently, an appointment will be given to all potential participants to attend a wellbeing clinic for baseline measures, linked to their diabetes clinic, within a week. A telephone call will be made to all willing participants to remind them of their appointment and ensure activation of the accelerometer.

**Fig. 1:**
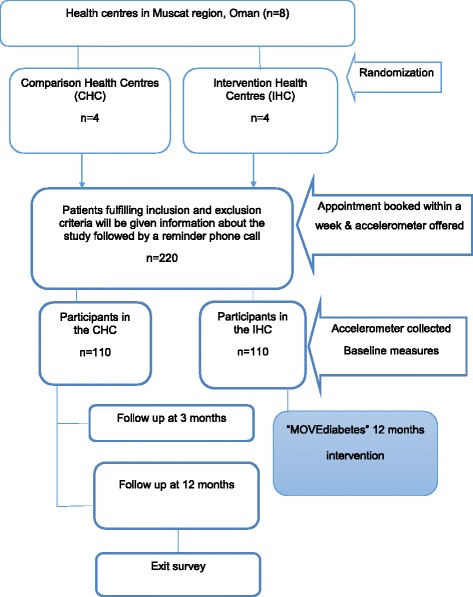
Participants flow chart

At the baseline visit the project officers will seek written informed consent and log any eligible individuals who decline participation (Fig. 1).

Recruitment will be monitored fortnightly and efforts to reduce loss to follow-up will be made. Participants not attending their appointment will be called to consider rescheduling their appointments. Reasonable travel costs will be reimbursed.

## Measures/assessment instruments

Baseline and follow-up data will be collected face to face and from the electronic health information system in the health centre (AlShifa system) (Table 1).

**Table 1:** Outcome measures

	Tool	When	
		Intervention	Comparison
Primary outcome			
GPAQ-MET-mins/week	Questionnaire	B, 3 F, 12 F	B, 3 F, 12 F
Accelerometer (activePAL^TM^)	Reporting step count, MET-min/week, and sitting time	B, 3 F, 12 F	B, 3 F, 12 F
Pedometer (YAMAX Digi-walker SW-200)	Reporting step counts	B, 3 F, 12 F	-
Secondary outcomes			
Socio-demographic data	Questionnaire	B	B
Height (m)	Stadiometer	B	B
Body weight (Kg)	Calibrated scales	B, 3 F, 12 F	B, 3 F, 12 F
Waist circumference (cm)	Tape measure	B, 3 F, 12 F	B, 3 F, 12 F
Lipid profile (mmol/l)	Blood test (venous fasted sample)	B, 3 F, 12 F	B, 3 F, 12 F
Blood pressure (mmHg)	Sphygmomanometer	B, 3 F, 12 F	B, 3 F, 12 F
^a^HbA1c (%)	Blood test (fasted sample)	B, 3 F, 12 F	B, 3 F, 12 F
Self-assessed general health	Questionnaire	B, 3 F, 12 F	B, 3 F, 12 F
Self-efficacy for PA	Questionnaire	B, 3 F, 12 F	B, 3 F, 12 F
Social support for exercise	Questionnaire	B, 3 F, 12 F	B, 3 F, 12 F
Cost analysis (description)	Detailed cost description	12 F	-
Exit survey	Questionnaire (participants and project officers)	12 F	-

(B = baseline; 3 F = 3 month follow-up, 12 F = 12 month follow-up)

^a^while blood collection for HbA1c at 12 month is mandatory, it is only done at baseline and 3 month if missing from the electronic health information system or recorded within more than 4 months prior to the measurement visits

## Intervention

The intervention group will receive the “MOVEdiabetes” personalised PA consultations, pedometer (YAMAX Digi-walker SW-200) to measure weekly step counts and WhatsApp messages (Table 2).

**Table 2:** MOVEdiabetes intervention components

Intervention visits	Weeks												
	0^a^	4	8^a^	12	16	20	24	28	32	36	40	44	48
Face to face physical activity consultations	→	↔	←										
Weekly WhatsApp step count	→	↔	↔										←
Monthly WhatsApp messages	→	↔	↔	↔	↔	↔	↔	↔	↔	↔	↔	↔	←

^a^After 7 days of PA recordings from accelerometers in the selected sub groups

### Face to face PA consultations

Recruited participants will be offered individual consultations (maximum 20 min) by the trained project officers on 3 occasions (0, 4 and 8 weeks) (Table 2). This will be undertaken a week after completing 7 days of accelerometer wear in the selected participants (accelerometer group) mostly in week 0 and 8.

The consultations aims to encourage participants towards achieving 150 min of PA per week (≥600MET-mins/week) at 12 months which has been demonstrated to be clinically effective in diabetes management. It is estimated that a step count of not less than 6000–7000 per day is required to achieve this goal [19]. Participants will be encouraged to increase their step counts gradually to achieve this goal.

PA programme design based on a theory and the behavior change techniques is widely proven to be effective [20, 21]. The theoretical framework underpinning the intervention in this study is the Health Belief Model, the Stages of Change Model and the Social Cognitive Theory [7]. The MOVEdiabetes personalised, multiple contact, intervention programme will also be based on several behavior change techniques based on the Abraham and Michie taxonomy [22] which includes (a) goal-setting for PA; (b) self-monitoring to achieve these goals; (c) frequent contact to provide accountability and sustain focus; (d) use of problem-solving to address goals and potential barriers to achieving them; and (e) emphasis on managing individual high-risk situations.

### Pedometer

Participants will be given a pedometer (YAMAX Digi-walker SW-200) at their baseline visit. Instructions on how to use the pedometer, how to record their daily steps and how to set daily step goals will be discussed by the project officers. Participants will be asked to set individual goals and fill in a daily step count to be submitted to the project officers in their respective health centres over a three month period and at 12 months.

### WhatsApp

Participants receiving the intervention will be asked to open and share a telephone WhatsApp application with the project officers in their health centre to facilitate the reporting of their step counts and get support during the intervention period. Additionally, monthly standardized physical activity motivational messages will be delivered through this telephone application. Participants are also invited to join a WhatsApp peer support group to share their experiences with other MOVEdiabetes participants.

## Process evaluation

Programme acceptability will be explored post-intervention via a brief exit questionnaire with all project officers and 50% of intervention participants randomly selected by random number tables generated in SPSSv21 by the principal investigator. The questionnaire will explore the extent initial expectations and motivations regarding the programme were met, engagement with the programme, acceptability (content, delivery and aims) of the approach e.g. if the intervention was tailored to be appropriate and realistic to the individual’s lifestyle, and elements of overall rating of the project including factors influencing willingness and ability to comply with the programme advice.

## Assessment of fidelity to protocol

- Managerial:
  - The project group will have monthly meetings to discuss issues regarding the physical activity consultations, and measurements to ensure their compliance to intervention protocol.
  - A telephone application (WhatsApp) will be used throughout the study period by project officers and the PI to manage the daily logistics and administrative queries.
  - Attendance sheets will be reviewed and discussed.
- Qualitative:
  - Audio-recording and transcription of 5 PA consultations randomly selected by random number tables generated in SPSSv21.
  - Crosschecking of 10% of PA consultation notes randomly selected by a recruited external assessor.
- Evaluative:
  - The brief exit survey will include questions on adherence to the protocol specifically for project officers.

## Sample size

To demonstrate a 50% between group difference in physical activity levels (MET-mins/week) over 12 months, to be detected at a power of 80%, and significance level of 5%, 128 participants would be required to complete the study (64 in each arm). Based on a drop-out rate of 20%, 154 participants would require to be recruited (77 in each arm). Assuming a recruitment rate of 70%, 220 potential eligible participants would need to be approached.

## Statistical analysis

The initial quantitative analysis will be an intention-to-treat analysis between the two cluster groups (intervention versus control) but secondary analyses will explore the effect of actual treatment received. The initial analysis will involve standard two-sample comparisons (parametric or nonparametric as dictated by the distribution of the data) looking at effect sizes at 3 and 12 months using t-tests or Mann-Whitney tests for differences in means as well as repeated measures or chi squared tests for differences in proportions. Differences by health centre will be explored and, if statistically significant will be entered in a mixed model as a random effect. The balance of characteristics between treatment and control arms will be tabulated and if differences are noted, adjustment will be made for these in linear regression models.

Results from the open-ended questions in the exit survey will be analysed thematically to identify the perceived acceptability of the intervention (content, delivery and aims) to the participants and project officers (health care providers).

## Discussion/rationale for current trial

Adults with T2D are known to have multiple comorbidities and are generally less active than the general population [23]. Behaviour change programmes targeting vulnerable populations are more effective than those targeting the population at large [24]. Additionally, use of technology namely phone applications (WhatsApp in this study) is a positive followup and monitoring tool to promote longterm PA [15].

PA on a regular basis improves metabolic, blood lipid profile control and quality of life [7]. Additionally, several studies have shown preventive effects of physical activity in individuals with T2D in lowering the risk of cardiovascular disease and premature death [25].

One advantage of the primary care setting in this study is its familiarity to potential participants. More importantly, when interventions are delivered in a clinical setting, desired outcomes are enhanced because of better medical endorsement and feelings of the intervention being an integrated part of care [26].

It is hoped that results from this study will enhance the evidence base for effective routes to increasing physical activity in inactive aduts with T2D; by assessing the impact of MOVEdiabetes intervention on PA levels; providing a platform (feasibility evidence) for the MOVEdiabetes intervention to be initiated in routine primary care clinics; increase understanding of participant engagement, barriers, opportunities; and examining cost related issues about intervention procedures in this clinical and cultural setting.

This study is of direct relevance to the ministry of health of Oman and has the potential to significantly enhance current government action on promoting physical activity. The study will equally assist in formulation and implementation of suitable plans of the National Health Policy Priorities: “5. To promote the health awareness of the community and establish a culture of healthy lifestyles” [27].

## Conclusion

It is hoped that this research will help to inform current policies and practices of the Omani Ministry of Health for the use of a culturally acceptable physical activity interventions as an integral part of care for clients with T2D within the primary health care setting.

## Abbreviations

BMI: Body Mass Index
CHC: Comparison Health Centre
GCC: Gulf Cooperation Council
GPAQ: Global Physical Activity Questionnaire
HbA1c: Glycated heamoglobin
IDF: International Diabetes Federation
IHC: Intervention health centre
IPAQ: International Physical Activity Questionnaire
MENA: Middle East and North Africa
PA: Physical activity
Scot-PASQ: Scottish Physical Activity Screening Questionnaire
WHO: World Health Organization
